# Giant Cell Tumor of the Distal Fibula Managed by an Autologous Proximal Fibula Graft

**DOI:** 10.7759/cureus.42620

**Published:** 2023-07-28

**Authors:** Soima Ali Muhammad, Afia Salman, Badaruddin Sahito, Jawad Ahmed

**Affiliations:** 1 Paediatrics and Child Health, Dow University of Health Sciences, Karachi, PAK; 2 College of Medicine, Dow University of Health Sciences, Karachi, PAK; 3 Orthopaedic Surgery, Civil Hospital Karachi, Karachi, PAK; 4 Internal Medicine, Dow University of Health Sciences, Karachi, PAK

**Keywords:** giant-cell tumor of bone, reconstruction, en bloc resection, giant-cell tumor, distal fibula

## Abstract

A giant cell tumor of the bone is among the most common bone tumors encountered by orthopedic surgeons. These benign and aggressive tumors are most commonly present around the knee joint; however, rare cases may involve the distal fibula. An 18-year-old man presented with a painless swelling of the lateral aspect of the left ankle. Clinical examination, radiologic evaluation, and biopsy confirmed the diagnosis of a giant cell tumor of the distal fibula. The patient was treated with resection of the distal fibula followed by reconstruction using an ipsilateral proximal fibula graft. The post-operative recovery was uneventful, and the patient was doing well on the last visit, one month after the intervention.

## Introduction

Giant cell tumor of the bone (GCTB) is one of the most common bone tumors that orthopedic surgeons encounter. GCTBs are benign, aggressive tumors that rarely metastasize, usually to the lungs. The GCTB is an osteolytic circumscribed mass characterized by neoplastic mononuclear stromal cells, macrophages, and osteoclast-like giant cells [1,2]. GCTB is a manifestation of altered gene expression caused by epigenetic modifications of histone proteins and clonal chromosomal aberrations. This genetic derangement results in overexpression of the RANK/RANKL signaling pathway, ultimately resulting in osteolysis of the bone involved [2,3]. GCTBs occur in individuals with mature skeletons, hence affecting adults, and involve the epiphysis and metaphysis of long bones. The tumor sites in the lower limb involve the area around the knee joint and the proximal and distal fibula. While the area around the knee joint accounts for the most common site for GCTBs, with more than 50% of cases, the distal fibula accounts for less than 1% of the GCTBs [2,4]. The distal fibula, along with the distal tibia, talus bone, and numerous supporting ligaments, form the ankle joint, which plays a pivotal role in bearing weight and maintaining equilibrium in the body. GCTB of the distal fibula vastly affects the integrity of the ankle joint [5].

GCTBs account for only 5% of the primary bone tumors in the West, whereas this incidence increases by up to 20% in Asia. 1.6 per 10,000,000 people per year are affected by GCTBs in the USA [2,6]. Environmental factors that predispose to these tumors have not been identified yet; however, people affected by Paget’s disease are at risk of developing multifocal GCTB. Since the disease occurs in skeletally mature populations, the median age being 35 years, children are rarely affected by GCTB. Moreover, the female population has a higher incidence of GCTBs. Despite their higher frequency in the adult female population, age and sex do not influence tumor localization [2]. Here, we present a case of a GCT of the distal fibula in an 18-year-old male, appearing as a swelling on the lateral aspect of the left ankle. 

## Case presentation

An 18-year-old male presented to the orthopedic department of Dr. Ruth K. M. Pfau, Civil Hospital Karachi, with a six-month history of painless swelling with an insidious onset on the lateral aspect of the left ankle. The patient had noticed a progressive increase in the size of the swelling. He reported no association between fever and weight loss. Family history and past medical history were not significant.

**Figure 1:**
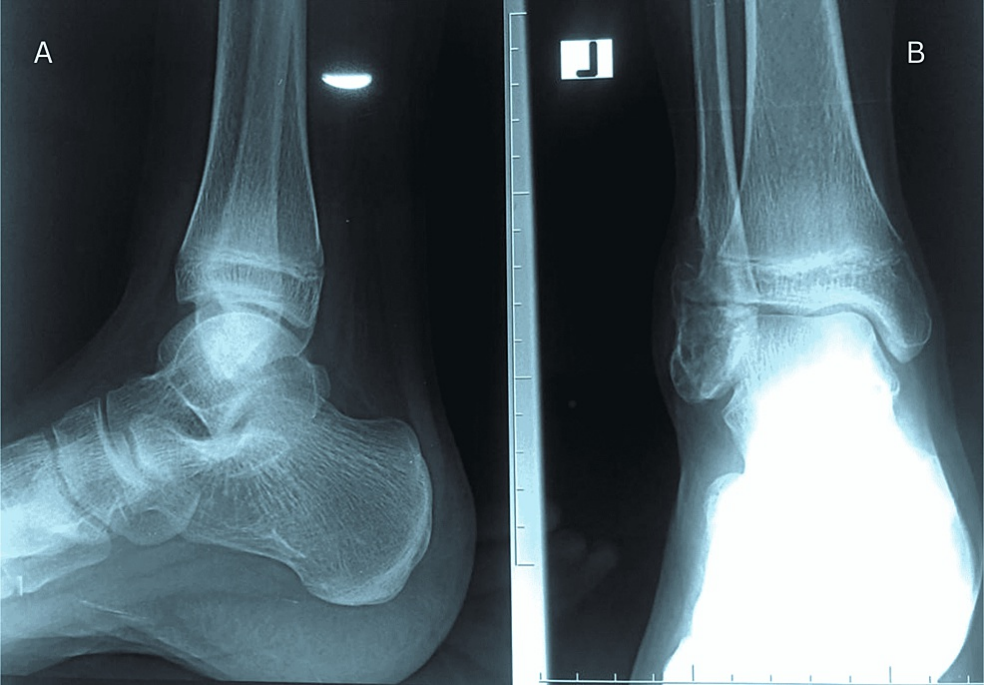
Pre-operative X-ray results. The lateral (A) and anteroposterior (B) views of the left ankle show an osteolytic balloon lesion of the lateral malleolus of the distal fibula. Septations within the tumor, a thin cortex, and a narrow zone of transition were also observed.

Local examination revealed a 4x5cm single fusiform lump on the lateral malleolus of his left ankle. The swelling was firm to hard in consistency, non-tender with overlying intact skin, and a freely mobile lump on palpation. The dorsalis pedis and posterior tibial artery were palpable. The range of ankle and subtalar joint movements was normal, with power in all tendons around the lateral malleolus of 5/5. Anteroposterior and lateral radiographs of the left ankle were taken, which showed the osteolytic raised lesion with an irregular appearance involving the distal fibula (lateral malleolus) with visible septations inside the lesions and a thin cortex. A narrow zone of transition with a geographic pattern was observed (Figure 1).

A magnetic resonance imaging (MRI) scan showed a hyperintense lesion on T2-weighted imaging involving the distal fibula and the normal peroneal tendon. Computed Tomography (CT) scan of the chest was negative for metastasis. A biopsy performed through a lateral incision was conclusive of GCT of the distal fibula. All routine hematological investigations (complete blood count, clotting studies, and vitamin K levels) were found to be normal.

Operative procedure

Distal fibula resection and reconstruction with the ipsilateral proximal fibula were planned, and the patient gave informed consent. 

**Figure 2:**
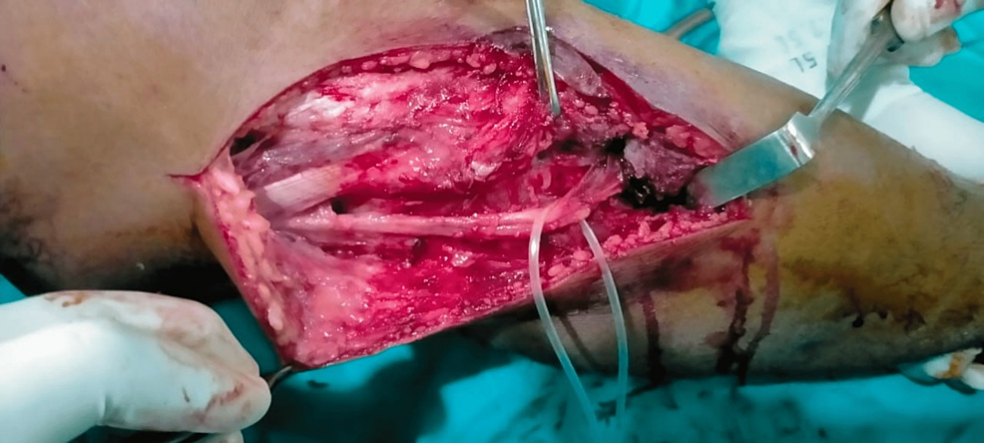
The incision was made on the lateral side over the distal fibula, including the biopsy scar. A fibula segment 2 cm superior to the tumor was dissected via osteotomy. The peroneal longus tendon was identified and split.

**Figure 3:**
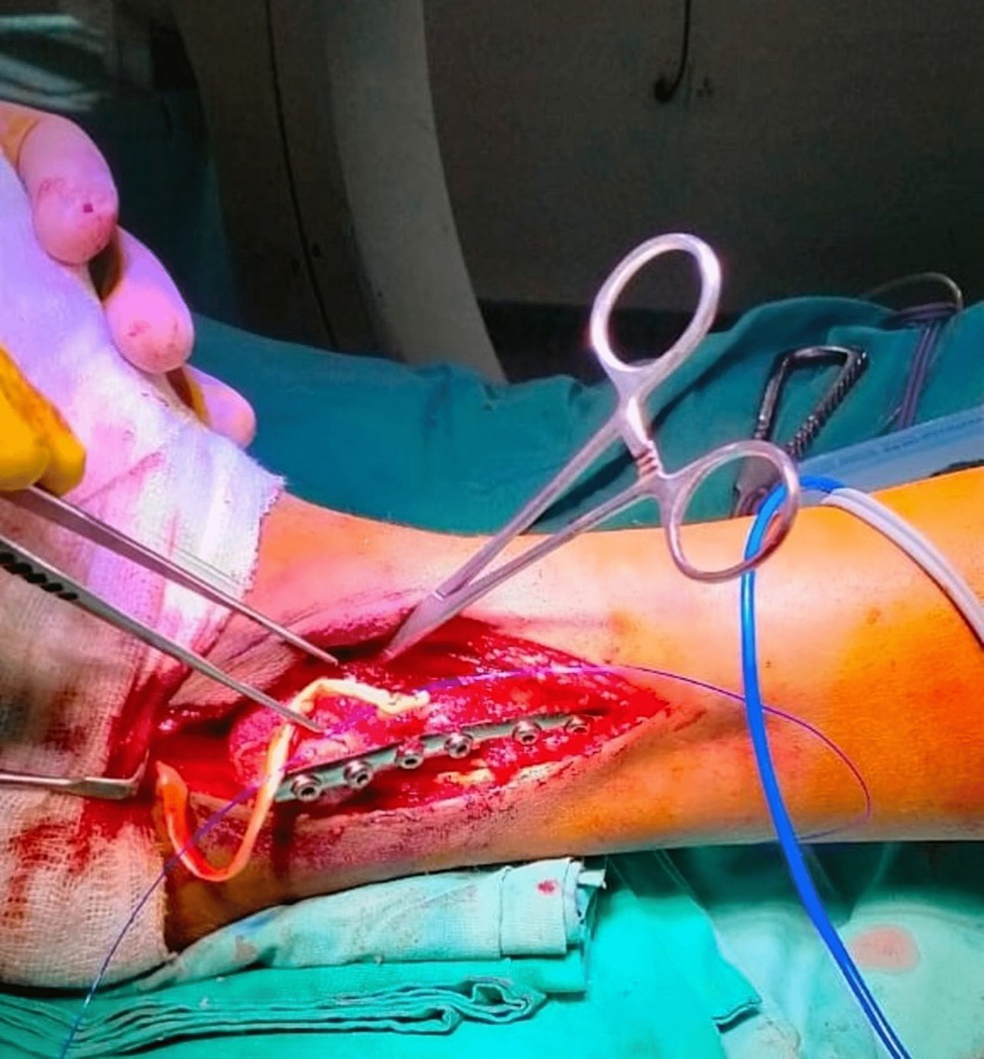
The bone graft from the proximal fibula was reverted and fixed at the distal fibula. A semi-tubular plate and a syndesmotic screw were also placed. Holes were made in the distal tibia and grafted fibula, and the peroneus longus tendon was passed through these holes and re-attached.

**Figure 4:**
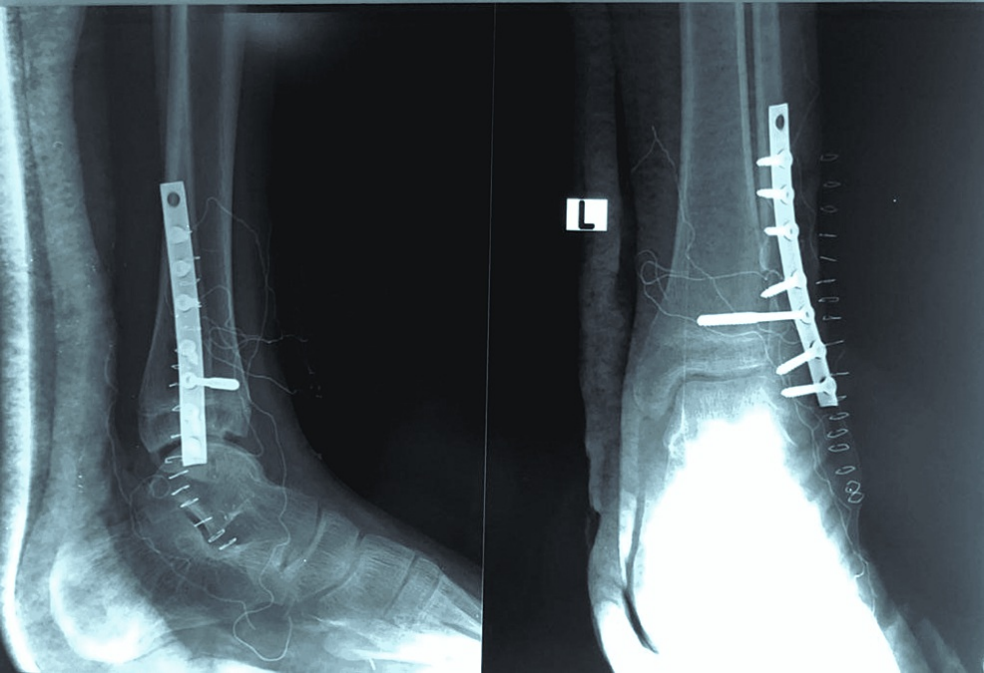
The lateral (right) and anteroposterior (left) views of the left ankle are shown. A bone graft from the proximal fibula, along with a semi-tubular plate and syndesmotic screw, was fixed in place where the tumor was dissected out.

The procedure was performed under general anesthesia in the lower extremities. After disinfecting the site, the incision was made in such a way as to include the biopsy scar on the lateral side of the fibula. An osteotomy was performed about two inches above the tumor, and the fibula was removed. Another incision was placed at the proximal end of the fibula, dissecting the skin and subcutaneous tissue and pointing to the biceps tendon. The common peroneal nerve was seen behind the proximal fibula and was separated and attached to the anterior side of the fibula (Figure 2). A proximal fibula graft of 6 cm was taken. To provide stability at the proximal fibula, a one-third semi-tubular plate, and a syndesmotic screw were placed. The peroneus longus tendon was identified and dissected; a drill hole was made in the distal tibia, and the tendon was pulled through this hole for re-attachment (Figure 3). The wound was closed, and the back slab below the knee was applied. A post-operative X-ray was also performed (Figure 4). The post-operative recovery was uneventful, and the patient was doing well on the last visit, one month after the intervention. 

## Discussion

In this case study, the patient presented with a six-month history of painless swelling with an insidious onset on the lateral aspect of the left ankle. The family history and past medical history of the patient were not significant, and the swelling was not associated with fever or weight loss. Local examination, pre-operative X-ray, MRI, and CT scan were performed before the surgical resection of the tumor. Post-operative imaging was performed to ensure adequate placement of a one-third semi-tubular plate and syndesmotic screw. At the patient’s one-week follow-up, the surgical site incision demonstrated adequate healing.

GCTBs are benign yet aggressive lesions that usually affect the femur, patella, tibia, and fibula, the bones around the knee joint. The distal femur and proximal tibia are the most frequently affected sites; however, the involvement of other bones is also seen. Multicentric GCTB may affect multiple areas simultaneously, although the occurrence is extremely rare [7,8]. Radiographic evaluation, such as an X-ray, computerized tomography (CT) scan, and magnetic resonance imaging (MRI), is crucial for the diagnosis, which is further confirmed by histopathologic examination. The classical presentation of tumors on radiographic imaging may include solid soft tissue, bone loss, cortical destruction, pathologic fracture of the involved bone, and soft tissue invasion [9].

A biopsy examination of the tumor exhibits large multinucleated giant cells with mononuclear spindle cells and monocytes with nuclei similar to those of large osteoclast-like giant cells. The mononuclear spindle cells undergo neoplastic changes and develop into giant cells, which resemble osteoclasts owing to the expression of receptor activator nuclear factor K-B. This is also accompanied by cystic degeneration, hemorrhage, mitotic figures, spindle cell stroma, and hemosiderin deposition, which is responsible for the gross reddish-brown appearance of the tumor [10]. 

The staging system for bone tumors was developed by Wolf et al. in 1996 based on three key elements. Histopathologic and radiologic findings determine the grade of the tumor (G); the tumor can be benign (G0), low-grade malignant (G1), or high-grade malignant (G2). The site of the tumor (T) is evaluated with reference to its natural anatomic compartment. T0 tumors are benign intra-compartmental, T1 tumors are aggressive intra-compartmental, whereas T2 tumors are extra-compartmental and extend beyond the cortex and articular cartilage. The tumors are also classified based on the absence or presence of metastasis (M), termed M0 and M1, respectively [11,12].

In our case, the diagnosis was made by X-ray imaging and MRI, which were further confirmed by biopsy. The radiologic examination revealed that the tumor was classified as a T1 or grade 2 tumor. The characteristic features that support the diagnosis include septations inside the lesion, thinning of the cortex, a geographic pattern, and a narrow zone of transition.

The treatment plan usually includes surgery; however, nonsurgical methods are also considered when surgery is not adequate. Nonsurgical methods include radiation therapy, embolization, and medications, which suppress tumor size and activity, thus preventing further bone damage. Medical management targets the RANK pathway and suppresses the osteoclastic activity of the tumor. The pharmaceutical agent administered in GCT is denosumab, which acts by reducing bone resorption. On the contrary, surgical treatment includes curettage, bone grafts, bone reconstruction, and artificial joints, depending upon the severity of bone and soft tissue damage [13,14]. In the presented case, the distal fibula was surgically resected, and bone reconstruction was performed, obtaining the bone graft from the ipsilateral proximal fibula. The surgical approach was favored, keeping in consideration the size of the lesion and the advantage of a screw and plate in providing adequate support to the involved region, which would have otherwise led to restricted mobility and impaired quality of life for the patient.

GCTs of the distal fibula are a rare occurrence, as evident by the fact that there are only seven studies to date that report the occurrence of GCTs involving the distal fibula [4,15-20]. These tumors are catastrophic and destroy the affected bone, thus affecting the mobility of the respective joint. In the case of GCTs of the distal fibula, movement at the ankle joint is limited by the tumor; thus, the patient cannot ambulate properly [4]. 

Bhowmick et al. have presented a case of a GCT of the right distal fibula with painful globular swelling on the lateral side and limited ankle mobility. The surgery steps were tailored to preserve ankle function, including excision of the bone fragment, extended curettage, and insertion of a bone graft obtained from the fibula proximal to the tumor site [20]. A similar case finding was reported by Eger et al. in which the tumor had not only damaged the distal fibula but also invaded the ankle joint, owing to which synovectomy was also performed along with tumor and bone resection. Bone reconstruction was performed a month after tumor resection using a bone graft from the iliac crest, and another revision was required five months after this surgery to correct the failed bone graft [15].

Vaishya et al. reported a case of a GCT of the left distal fibula, presenting as non-tender swelling on the lateral side without compromising the range of movements at the ankle joint. In this case, phenol was used for chemical cauterization, and extended curettage of the tumor and resection of the lateral wall of the distal fibula were performed, followed by reconstruction using a bone graft from the iliac crest. Concurrently, we present a case with a similar clinical presentation; however, surgical resection was the ultimate choice of treatment, and curettage and chemical cauterization were not performed [4]. Another interesting case study published by Rangaswamy et al. elaborates on a bleeding lesion at the lateral malleolus of the left leg that manifested as a recurrence of a GCT of the distal fibula that was treated by curettage a year before. The tumor was resected, and mesh and screws were used to stabilize the joint [18]. 

The Campanacci grading system has been utilized for the classification of GCT based on the radiographic appearance. Grade I, or latent lesion, is characterized by an intact cortex and a distinct margin. Grade II, or active lesion, is represented by thinning and moderate expansion of the cortex and a well-defined margin without any radiopaque rim. Grade III, or aggressive lesion, exhibits destruction of the cortex and indistinct borders [14]. 

A case series of GCTs by Fain et al. includes one case of GCT of the distal fibula involving the metadiaphysis. The tumor was classified as Campanacci stage III on biopsy. Surgery was performed to excise the tumor, and no recurrence was recorded within five years [16]. Another case series by Jamshidi et al. presented individuals suffering from GCT of the distal fibula accompanied by pain and swelling of the lateral aspect of the ankle. The bone allograft for reconstruction was obtained from the tissue bank and inserted after the resection of the bone and tumor [17]. The post-operative recovery of our patient was eventless. 

## Conclusions

The present case report of Campanacci grade II GCT of the distal fibula on the lateral side of the left ankle elaborates that the tumor had no other manifestations except for a mobile fusiform swelling. No other remarkable findings were recorded upon examination. The initial diagnosis was made using X-ray and MRI modalities, and a CT scan of the lungs was also performed to rule out metastasis. The diagnosis was further confirmed via biopsy, and a treatment plan was devised based on en bloc resection of the tumor and reconstruction with a bone graft obtained from the ipsilateral proximal fibula. The recovery phase was uneventful. In contrast with other case presentations, curettage was not performed in this case.
